# Total Neoadjuvant Therapy Versus Conventional Chemoradiotherapy in Rectal Cancer: Impact on Tumor Regression Grade and the Predictive Value of CEA

**DOI:** 10.3390/medicina62010226

**Published:** 2026-01-22

**Authors:** Aikaterini Sarafi, Aikaterini Leventi, Klaountia Athitaki, Konstantinos Stamou, Ioannis Papaconstantinou, Dimitrios Korkolis

**Affiliations:** 1 Department of Surgery, General Hospital of Chania, 733 00 Chania, Greece; ktrnsrf@gmail.com; 2 Department of Surgical Oncology, Oncology Hospital Saint Savvas, 115 22 Athens, Greece; ksleventi@yahoo.com (A.L.); athitaki.c@gmail.com (K.A.); 3 Department of Surgery, Mitera Hospital, 151 23 Athens, Greece; kostas@stamou-surgery.gr; 4 Department of Surgery, Aretaieio Hospital, 115 28 Athens, Greece; johnpapacon@hotmail.com

**Keywords:** total neoadjuvant therapy, chemoradiation, tumor regression grade, rectal cancer, CEA

## Abstract

*Background and Objectives*: The introduction of total neoadjuvant therapy (TNT) in the preoperative stage has been associated with improved oncological outcomes. However, TNT may lead to tissue fibrosis and be accompanied by increased difficulty during surgery. Additionally, predicting tumor response to neoadjuvant therapy is crucial for identifying patients who may achieve a complete pathological response (pCR) or qualify for organ-preserving strategies. The aim of this study is to evaluate the effect of TNT versus conventional chemoradiotherapy (CRT) on tumor regression grade (TRG) and the association between preoperative carcinoembryonic antigen (CEA) levels and good tumor response. A secondary endpoint is to investigate the effect of TNT on surgical difficulty, using indirect indicators like the quality of total mesorectal excision (TME), circumferential resection margin (CRM), and achievement of R0 resection. *Materials and Methods*: This is a retrospective, single-center study including 93 patients with locally advanced rectal cancer who received either TNT (*n* = 43) or CRT (*n* = 50). *Results*: The TNT group, compared to the CRT group, demonstrated a significantly higher rate of pCR (TRG0) (37.2% vs. 18%, *p* = 0.038) and good tumor regression (TRG 0–1) (53.5% vs. 28%, *p* = 0.019). Furthermore, patients with CEA < 5 ng/mL showed significantly higher rates of good tumor response (TRG 0–1) compared to those with CEA ≥ 5 ng/mL (45.3% vs. 16.7%, *p* = 0.032). When further categorized by treatment type, CEA levels did not demonstrate statistically significant differences Lastly, increased surgical difficulty could not be established, as no significant differences were observed in terms of positive CRM rates, R0 resection, and TME quality between groups. *Conclusions*: TNT was associated with improved TRG scores compared to CRT without increasing surgical difficulty. Lower pre-treatment CEAs were linked to better tumor response, irrespective of the type of treatment. These findings support the oncological benefit of TNT and suggest that CEA may have some predictive value for treatment response.

## 1. Introduction

In recent years, the treatment paradigm for rectal cancer has shifted with the introduction of total neoadjuvant therapy (TNT), which incorporates systemic chemotherapy into the preoperative phase. This approach has been endorsed by major clinical guidelines, including those of the NCCN [1], based on results from large, randomized trials such as RAPIDO and PRODIGE-23 [2,3]. Both the RAPIDO trial short-course radiotherapy (5 × 5 Gy), followed by consolidation chemotherapy with CAPOX or FOLFOX4—and the PRODIGE-23 trial induction chemotherapy with FOLFIRINOX prior to long-course chemoradiotherapy (50.4 Gy with concurrent capecitabine)—demonstrated significantly higher rates of complete pathological response (ypT0N0) and improved distant disease control in patients receiving TNT compared to standard CRT. Simultaneously, TNT is playing a pivotal role in expanding access to nonoperative management strategies, particularly in patients who achieve a clinical complete response (cCR). In such cases, the “watch and wait” approach, increasingly supported by evidence, offers the potential to avoid radical surgery and preserve anorectal function without compromising oncological safety. This represents a significant shift toward personalized, response-adapted care in rectal cancer.

Although the majority of patients treated with TNT experience substantial tumor regression, approximately 20% demonstrate minimal or no response, deriving limited benefit from the intensified preoperative regimen and the extended interval to surgery [4]. As a result, there is growing interest in identifying predictive tools that can help clinicians tailor TNT to patients who are most likely to respond. Some studies, such as those by Cheong et al. [5] and Chapman et al. [6], have highlighted the potential role of carcinoembryonic antigen (CEA) as a predictive biomarker for tumor response, although evidence remains limited, and further validation in large cohorts is required.

Beyond oncologic response, TNT may impact surgical complexity. Fibrotic changes induced by neoadjuvant treatment can lead to the distortion of normal pelvic anatomy, loss of tissue planes, and reduced mobility of mesorectal structures—factors that increase the technical difficulty of total mesorectal excision (TME). Several surrogate markers have been proposed in the literature, including TME specimen quality (assessed using Quirke criteria), CRM involvement, R0 resection status, intraoperative blood loss, total operative time, duration of pelvic dissection, and the incidence of anastomotic leakage, as indicators of surgical complexity [7,8]. While the RAPIDO and PRODIGE studies included secondary analyses of these surgical factors, no significant differences were observed between TNT and CRT groups—though these were not primary endpoints in either trial [2,3].

The primary aim of our study is to evaluate the effect of TNT compared to conventional preoperative chemoradiotherapy (CRT) on tumor regression grades (TRGs). The secondary objectives were to evaluate the association between preoperative carcinoembryonic antigen (CEA) levels and tumor response to neoadjuvant therapy and to explore whether TNT is associated with increased surgical difficulty, using surrogate pathological indicators such as total mesorectal excision (TME) quality, circumferential resection margin (CRM) status, and R0 resection rates.

## 2. Materials and Methods

This is a retrospective, single-center, observational cohort study analyzing pathological and prognostic parameters in patients with locally advanced rectal cancer who underwent neoadjuvant therapy followed by surgical resection at the Department of Surgical Oncology of the General Anticancer Oncological Hospital of Athens “Agios Savvas”, Greece. Clinical data recorded in the institutional database and patient medical records were analyzed following the acquisition of informed consent for investigational and educational purposes. Each patient was assigned a unique identification code, and their data were transcribed into an anonymized pro forma by a designated researcher who was blinded to the data analysis.

The study population consisted of patients diagnosed with locally advanced rectal adenocarcinoma, for whom treatment decisions were made after discussion with the local Gastrointestinal Oncological MDT, according to the department’s clinical protocol, which aligns with current international guidelines (ESMO, NCCN) [1,9]. As this was a pragmatic, real-world clinical study, the criteria for treatment selection reflected current oncological practice at our institution. Eligible patients received either conventional neoadjuvant chemoradiotherapy (CRT) or total neoadjuvant therapy (TNT) and subsequently underwent curative surgical resection between 1 January 2023 and 1 January 2025. Inclusion criteria were age ≥ 18 years at the start of treatment, diagnosis of locally advanced rectal cancer (stage ≥ cT3 and/or cN+, M0), completion of neoadjuvant therapy (CRT or TNT) as determined by multidisciplinary tumor board decision, and undergoing total mesorectal excision (TME) with curative intent within the defined study period. Exclusion criteria were the presence of distant metastases (TNM stage IV), early-stage rectal cancer (cT1–T2, N0), emergency surgical interventions (e.g., for perforation, bleeding, or complete obstruction), pregnancy at the time of diagnosis or treatment, and non-curative (palliative or cytoreductive) surgery.

Prior to treatment, all patients underwent a complete clinical, laboratory, and imaging work-up for staging, including contrast-enhanced CT of the chest, abdomen, and pelvis. Additionally, all patients underwent high-resolution pelvic MRI with a rectal protocol before and 4–6 weeks after neoadjuvant therapy (timing differed depending on TNT regimen: induction or consolidation) to assess local tumor stage and treatment response. The therapeutic approach was individualized based on the decisions of the multidisciplinary tumor board, considering imaging findings, histopathological characteristics, and clinical status, in accordance with current international guidelines (ESMO 2025, NCCN 2025) [1,9].

Patients with intermediate-risk tumors (cT2 with positive nodes or cT3N0–1) located in the upper rectum were eligible for upfront surgery, whereas tumors located in the middle and distal rectum received neoadjuvant CRT or SCRT prior to TME with adjuvant chemotherapy, in accordance with guideline recommendations. Patients with high-risk features (T4, cN2, threatened mesorectal fascia [mrMRF+], EMVI+, or positive lateral pelvic nodes) received TNT, either as induction or consolidation, followed by TME, according to international guidelines and under the supervision of the multidisciplinary tumor board. At the time this study was conducted, TNT was given following the RAPIDO protocol for Short-Course CRT and the OPRA protocol for Long-Course CRT [2,10].

All surgical procedures were performed 8 to 12 weeks after completion of neoadjuvant treatment, following standard TME principles. All surgical procedures were performed according to standardized total mesorectal excision (TME) principles by senior consultant surgeons in the department, with subspecialty interest in colorectal surgery and formal training in TME techniques. The choice of surgical approach (open or minimally invasive) was made by the operating surgeon based on tumor characteristics and patient-related factors. Surgical technique and perioperative management protocols remained consistent throughout the study

In total, 93 patients were enrolled in the study. For comparative analysis, the cohort was retrospectively divided into two distinct treatment groups: CRT group: patients who received conventional neoadjuvant chemoradiotherapy (CRT), either short-course or long-course (*n* = 50); and TNT group: patients treated with total neoadjuvant therapy (TNT) (*n* = 43).

Patients in the CRT group received either short-course radiotherapy (SCRT) or long-course chemoradiotherapy (LCRT) prior to surgery. LCRT was delivered using volumetric modulated arc therapy (VMAT), with daily image guidance using low-dose computed tomography (IGRT) to ensure accurate treatment delivery. A total tumor dose of 50 Gy was administered in 25 fractions (2 Gy per fraction), concurrently with oral capecitabine. Patients in the SCRT group received 25 Gy delivered in 5 fractions of 5 Gy each, following standard SCRT protocols. Radiation planning, setup, and delivery techniques were consistent throughout the study period.

All surgical specimens were evaluated by two experienced gastrointestinal pathologists. Tumor regression grade (TRG), circumferential resection margin (CRM) status, R0 resection status, and mesorectal excision quality were assessed according to validated and standardized criteria (Ryan TRG system and Quirke classification) [11,12]. Pathologists were blinded to the neoadjuvant treatment regimen during assessment.

All specimens in our study were classified according to Ryan Grade score, and, for analysis purposes, they were also grouped as good response, including TRG 0 and 1, and bad response, including TRG 2 and 3.

As far as the Quirke TME criteria for specimen quality is concerned (Table 1), they were originally used to evaluate the quality of the mesorectal excision and have been associated with reduced local recurrence and improved outcomes [12]. When total mesorectal excision (TME) is performed using a standardized technique by an experienced and consistent surgical team, like in our study, the pathological assessment based on the Quirke criteria may serve as an indirect surrogate marker of procedural complexity [13]. In this context, a technically demanding case is more likely to yield suboptimal mesorectal quality despite uniform surgical execution, thereby reflecting intrinsic anatomical or oncological challenges rather than variability in surgical performance.

Carcinoembryonic antigen (CEA) is frequently used as a prognostic biomarker for response to neoadjuvant therapy, as elevated levels have been associated with lower rates of tumor regression following preoperative chemoradiotherapy [5,6]. Measurement of preoperative CEA provides a simple, noninvasive indicator that may help assess tumor biological aggressiveness and guide personalized treatment decisions. In this study, only preoperative CEA levels (measured one day before surgery, following completion of neoadjuvant therapy) were available for analysis and were therefore recorded and studied as a potential predictive biomarker of complete pathological response (pCR) within the context of personalized oncologic treatment planning.

### Statistical Analysis

Continuous variables are expressed as mean ± standard deviation or as median (interquartile range) in cases of non-normal distribution. Categorical variables are presented as frequency (*n*) and percentage (%). The normality of distribution was assessed using the Kolmogorov–Smirnov test.

Comparisons between the two treatment groups for demographic and clinical parameters and assessment indices were performed using the independent samples *t*-test and the χ^2^ test, as appropriate.

A multivariable logistic regression model was applied to examine the effect of treatment and carcinoembryonic antigen (CEA) level on tumor regression grade (TRG), adjusting for relevant demographic and clinical covariates. All assumptions for regression analysis (homoscedasticity, linearity, normal distribution, and independence of residuals, plus absence of multicollinearity among independent variables) were assessed.

All analyses were performed using SPSS version 21.0 (IBM Corporation, Somers, NY, USA). All tests were two-sided, and a *p* value < 0.05 was considered statistically significant.

## 3. Results

To ensure a reliable comparison between the two treatment groups (CRT and TNT), sample homogeneity was assessed for key baseline characteristics that could potentially influence the pathological response and overall prognosis. The variables examined included age, sex, body mass index (BMI), and tumor anatomical location. These factors are known to be associated with the response to neoadjuvant therapy, likelihood of complete tumor regression, and technical complexity of surgical resection.

The statistical analysis of the above variables showed that no statistically significant differences were observed between the two groups with respect to age (*p* = 0.624), sex (*p* = 0.064), BMI (*p* = 0.873), and tumor location (*p* = 1.00).

### 3.1. Tumor Regression Grade

Tumor regression differed between the treatment groups. TRG 0 was observed in 37.2% of TNT patients versus 18% in the CRT group (*p* = 0.038). TRG 1 occurred in 16.2% versus 10% and TRG 2 in 16.2% versus 32% (*p* = 0.040) of patients in the TNT and CRT groups, respectively. TRG 3 was reported in 30.3% of the TNT group compared with 40% in the CRT group.

**Figure 1:**
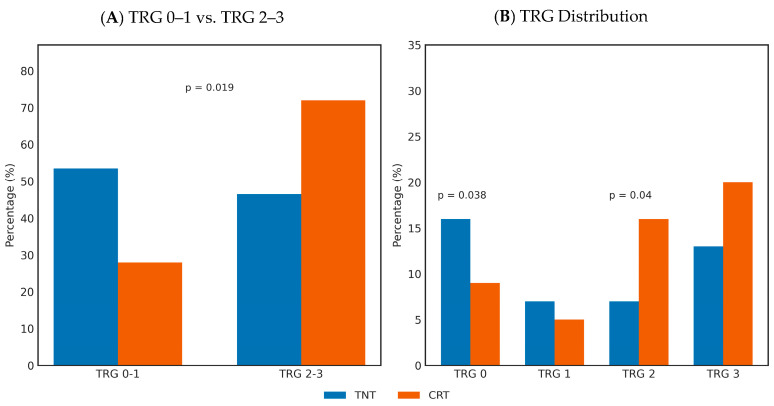
Tumor response to neoadjuvant treatment. Bar charts illustrating (**A**) combined TRG categories (TRG 0–1 vs. TRG 2–3) and (**B**) the distribution of individual TRG categories (TRG 0, 1, 2, and 3) in patients treated with total neoadjuvant therapy (TNT) versus chemoradiotherapy (CRT). Values are presented as percentages. Statistically significant differences were observed for the comparison between TRG 0–1 and TRG 2–3, as well as among individual TRG categories, where indicated.

When tumor regression was defined as complete or near-complete (TRG 0 or 1), the proportion of patients achieving this outcome was significantly higher in the TNT group compared with the CRT group (53.5% vs. 28.0%; *p* = 0.019), as presented in Figure 1.

Using a multiple logistic regression model with the enter method, we evaluated the effect of treatment type on achieving TRG 0–1, while adjusting for demographic and clinical covariates. The model met all assumptions regarding normality, homoscedasticity, the absence of influential outliers, and lack of multicollinearity.

The regression model was statistically significant, χ^2^ (5) = 11.40, *p* = 0.044, explaining 15.6% of the variance in the probability of achieving TRG 0–1 (Nagelkerke R^2^ = 0.156), with 65.6% of cases correctly classified.

The treatment type was found to have a statistically significant effect on the likelihood of achieving TRG 0–1 (*p* = 0.026), whereas age, sex, BMI, and tumor location were not significantly associated (*p* > 0.05). As shown in Table 2, patients treated with TNT were 2.8 times more likely to achieve complete or near-complete tumor regression (TRG 0–1) compared with those treated with CRT (*p* = 0.026).

### 3.2. Surgical Quality Assessment

Due to the retrospective nature of this study, the quality of the mesorectal excision, the circumferential resection margin (CRM), and the adequacy of R0 resection were evaluated based on pathological reports. Assessing these parameters provided additional insight into whether the type of neoadjuvant therapy (CRT vs. TNT) affected not only the oncologic response but also the technical difficulty and quality of the surgical procedure.

**Figure 2:**
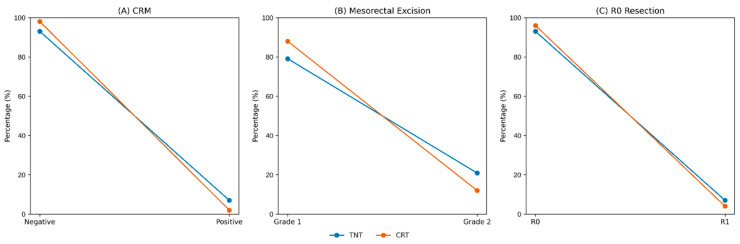
Surrogate markers of surgical difficulty according to treatment group. Dot-and-line plots comparing (**A**) the circumferential resection margin (CRM) status (negative vs. positive), (**B**) quality of mesorectal excision (Grade 1 vs. Grade 2), and (**C**) resection margin status (R0 vs. R1) between patients treated with total neoadjuvant therapy (TNT) and chemoradiotherapy (CRT). Values are presented as percentages. No statistically significant differences were observed between groups.

No statistically significant differences were observed between the TNT and CRT groups in terms of positive CRM rates (7% vs. 2%, *p* = 0.332) or R0 resection rates (7% vs. 4%, *p* = 0.659). A trend toward poorer mesorectal excision quality was noted among patients who received TNT, though this difference was not statistically significant (20.9% vs. 12%; *p* = 0.271) (Figure 2).

### 3.3. Prognostic Value of Cea

Overall, a significant association was observed between preoperative CEA levels and pathological tumor response. Patients with CEA < 5 ng/mL demonstrated higher rates of good response (TRG 0–1) compared with those with CEA ≥ 5 ng/mL (45.3% vs. 16.7%; *p* = 0.032, Fisher’s exact test). The odds ratio (OR) for good response among patients with low CEA was 4.15 (95% CI, 1.1–15.5).

**Figure 3:**
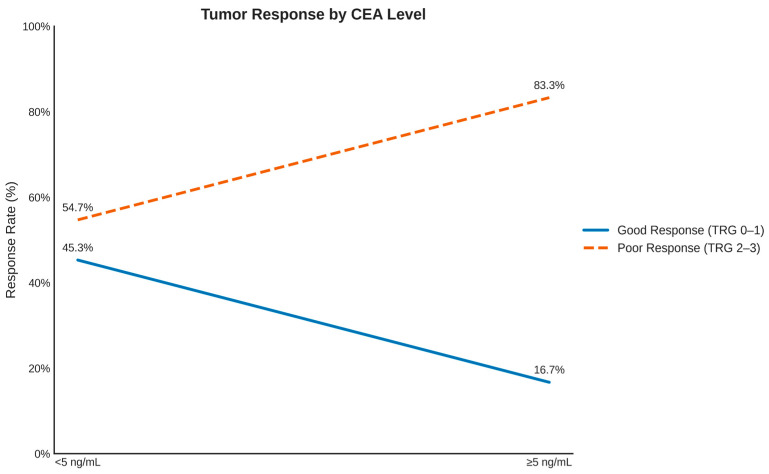
Tumor response in relation to CEA levels. Dot-and-line plot comparing good tumor response (TRG 0–1; solid line) and poor tumor response (TRG 2–3; dotted line) according to preoperative carcinoembryonic antigen (CEA) levels (<5 ng/mL vs. >5 ng/mL). Percentage values are displayed for each point. Patients with CEA < 5 ng/mL demonstrated a significantly higher likelihood of good response compared with those with elevated CEA levels (OR 4.15; 95% CI 1.1–15.5; *p* = 0.032).

The specificity of CEA was high (91.8%; 95% CI, 77.0–97.9%), whereas sensitivity was relatively low (26.8%; 95% CI, 16.2–40.5%), indicating that normal CEA is a strong negative predictor of poor response. The positive predictive value (PPV) was 83.3% (95% CI, 57.8–95.6%), and the negative predictive value (NPV) was 45.3% (95% CI, 33.9–57.2%) (Table 3, Figure 3).

#### 3.3.1. Subgroup Analysis: TNT Group

Among patients who received total neoadjuvant therapy (TNT), the association between preoperative CEA and the pathological response followed a similar trend as the overall cohort but did not reach statistical significance (*p* = 0.222, Fisher’s exact test). Patients with CEA < 5 ng/mL achieved higher rates of good response (TRG 0–1) than those with CEA ≥ 5 ng/mL (58.3% vs. 28.6%), with an OR = 3.5 (95% CI, 0.6–20.5).

Specificity remained high (91.3%; 95% CI, 70.5–98.5%), while sensitivity was low (25%; 95% CI, 10.0–49.4%). PPV was 71.4% (95% CI, 30.2–94.9%), and NPV was 58.3% (95% CI, 40.9–74.0%).

#### 3.3.2. Subgroup Analysis: CRT Group

In the CRT group, a similar but not significant trend was observed between preoperative CEA and tumor regression (*p* = 0.148, Fisher’s exact test). Patients with CEA < 5 ng/mL showed higher rates of good response (33.3%) compared with those with CEA ≥ 5 ng/mL (9.1%), with an OR = 5.0 (95% CI, 0.6–43.4).

CEA specificity remained high (92.9%; 95% CI, 64.2–99.6%), while sensitivity was modest (27.7%; 95% CI, 14.8–45.4%). PPV was 90.9% (95% CI, 57.0–99.5%) and NPV was 33.3% (95% CI, 19.6–50.3%).

#### 3.3.3. Multivariable Analysis

A multiple logistic regression model (enter method) was used to explore the independent effect of CEA on TRG 2–3, while adjusting for demographic and clinical variables. Model diagnostics confirmed assumptions of normality, homoscedasticity, and the absence of multicollinearity.

The model was statistically significant, χ^2^ (5) = 18.75, *p* = 0.005, explaining 24.5% of the variance in TRG 2–3 outcomes (Nagelkerke R^2^ = 0.245), correctly classifying 64.5% of cases.

CEA (*p* = 0.018), sex (*p* = 0.042), and treatment type (*p* = 0.039) were independent predictors of poor response (TRG 2–3), whereas age, BMI, and tumor location were not. Patients receiving CRT had 2.7 times higher odds of TRG 2–3 compared with those receiving TNT, and patients with CEA ≥ 5 ng/mL had 6.3 times higher odds of poor response, as shown in Table 4.

In the TNT subgroup, logistic regression showed a borderline significant model, χ^2^ (5) = 10.20, *p* = 0.070 (Nagelkerke R^2^ = 0.282), correctly classifying 72.1% of cases. None of the predictors reached statistical significance, although trends were noted for younger age, female sex, lower BMI, distal tumor location, and low CEA (<5 ng/mL). Patients with low CEA were approximately five times more likely to achieve a good response (OR = 5.33; 95% CI, 0.70–40.54; and *p* = 0.105).

In contrast, for patients treated with CRT, the logistic regression model was not statistically significant, χ^2^ (5) = 6.57, *p* = 0.254 (Nagelkerke R^2^ = 0.177), though it correctly classified 78% of cases. While CEA was not a significant independent predictor (*p* = 0.141), patients with CEA ≥ 5 ng/mL were approximately 6.6 times more likely to exhibit poor tumor regression (TRG 2–3). No other variable demonstrated statistical significance (*p* > 0.05). These findings suggest a possible association between elevated CEA and poor pathological response following CRT, which warrants further investigation in larger cohorts.

## 4. Discussion

The present study investigated the association between the type of neoadjuvant treatment (TNT versus conventional chemoradiotherapy, CRT) and pathological response (TRG) in patients with locally advanced rectal cancer. The objective was to assess the relative efficacy of these therapeutic approaches in achieving complete or near-complete tumor regression, as well as to explore the potential influence of pre-treatment CEA levels on pathological response.

Our study demonstrates that TNT is associated with substantially improved pathological tumor regression compared with conventional CRT. The higher rates of complete response (TRG 0: 37.2% vs. 18%, *p* = 0.038) and lower rates of inadequate regression (TRG 2: 16.2% vs. 32%, *p* = 0.040) align with contemporary evidence from major randomized trials. RAPIDO reported a pCR rate of 28% with TNT versus 14% with standard CRT [2], while PRODIGE-23 showed an increase from 11.7% to 27.5% with the addition of induction chemotherapy [3]. These convergent findings support the hypothesis that intensified systemic therapy may enhance tumor chemosensitivity, reduce micrometastatic burden, and prime the tumor microenvironment for an improved response to chemoradiation.

Multivariate analysis confirmed treatment modality as the only independent predictor of favorable regression (TRG 0–1), with TNT increasing the likelihood of good response nearly threefold (OR = 2.78, 95% CI: 1.13–6.83, and *p* = 0.026). No significant associations were observed with age, sex, BMI, or tumor location (*p* > 0.05). While prior studies have suggested potential links between BMI or distal tumor location and response [14,15], our findings raise the hypothesis that TNT may attenuate the influence of traditional anatomical or demographic predictors by exerting a dominant biological effect. Although, it is important to acknowledge that the modest sample size may have limited the power to detect smaller effects of other clinical or demographic factors, and, therefore, larger multicenter cohorts are needed to validate whether patient-level factors retain prognostic relevance in the TNT era.

Concerns regarding the impact of TNT on surgical difficulty remain clinically relevant. Although a trend toward inferior TME quality was observed in the TNT group (20.9% vs. 12%), this difference was not statistically significant, and neither CRM positivity nor R1 resection rates differed between groups. These findings are consistent with RAPIDO, which reported the deterioration in specimen quality following TNT but no compromise in CRM or R0 resection rates [16]. Conversely, the multicenter analysis by Lin et al. found no significant differences in complete TME or negative CRM rates between TNT and CRT [17].

Qualitative evaluations and published reports provide additional support for these observations: researchers using a video-based assessment were surgeons blinded to the original treatment and assigned difficulty scores to recorded operative procedures. TNT cases had higher scores (mean VAS 4.6) compared with LCRT (3.2) and upfront surgery (4.1), and surgeons were able to correctly identify TNT cases more frequently (71%), suggesting subtle visual or tissue changes, such as fibrosis or edema, that distinguish these cases [18]. Similarly, a nationwide survey of Dutch surgeons reported that 65% observed changes in tissue texture and boundaries, 47% noted difficulty identifying anatomical planes, and 32% experienced increased intraoperative bleeding during surgeries following TNT [19]. These observations support the hypothesis that TNT modifies the intraoperative landscape but that surgical expertise in high-volume centers mitigates these effects, preserving oncologic safety.

A notable finding of this study is the association between post-treatment CEA levels and pathological response. Patients with CEA < 5 ng/mL achieved significantly higher rates of TRG 0–1 (45.3% vs. 16.7%, *p* = 0.032), with an OR of 4.15 (95% CI: 1.1–15.5). This association persisted after adjustment for confounders, suggesting that CEA may serve as a dynamic biomarker of treatment sensitivity. The high specificity (91.8%) of elevated CEA for poor response raises the hypothesis that persistent elevation reflects biologically resistant disease. Clinically, this may support closer surveillance, a shorter interval to re-staging, or alternative therapeutic considerations

These findings are consistent with Yang et al., who reported that post-CRT CEA < 2.61 ng/mL predicted pCR with a sensitivity of 76.0% and specificity of 58.4% [20], and with Moureau Zabutto et al., who identified pre-CRT CEA < 5 ng/mL as an independent predictor of complete response (*p* = 0.019) [21].

Notably, major TNT trials (RAPIDO, PRODIGE-23, and CAO/ARO/AIO-12) have not incorporated CEA into predictive modeling [2,3,22], highlighting an opportunity for future prospective research. Dynamic CEA trends, as explored in the FOWARC trial [23], may offer even greater predictive accuracy than single time-point values.

When analyzed by treatment subgroup, statistical significance was lost, likely due to sample size limitations. In the TNT cohort, patients with CEA < 5 ng/mL showed a threefold higher likelihood of good response (58.3% vs. 28.6%, OR = 3.5, and *p* = 0.222), with a similar trend observed in the CRT group (*p* = 0.148, OR = 5.0). However, the consistent directional trends support the hypothesis that CEA may function as a treatment-agnostic biomarker of response.

Several limitations should be acknowledged. The retrospective, single-center design limits the control over confounding variables and introduces potential selection and recording bias. Although CEA was evaluated as a prognostic biomarker, the systematic documentation of baseline values and relative changes was incomplete, limiting interpretability. Treatment heterogeneity—including variation in CRT regimens (short- or long-course) and TNT protocols—along with missing data on pre-treatment CEA levels, may have affected the internal validity and intergroup comparability. These reflect the pragmatic and real-world nature of the study, mirroring the variability inherent in clinical practice. In addition, surgical complexity was assessed indirectly through surrogate histopathological indicators, such as CRM status and TME quality, without objective intraoperative parameters (e.g., operative time, blood loss), reducing the precision of the surgical outcome assessment. Finally, the modest sample size, particularly in subgroup analyses, limited the statistical power and may have obscured clinically relevant trends

## 5. Conclusions

Our findings support CEA as a prognostic biomarker for pathological response after neoadjuvant therapy in rectal cancer and confirm that total neoadjuvant therapy is associated with higher rates of pCR and favorable tumor regression. However, potential technical challenges related to therapy-induced fibrosis should be considered during multidisciplinary planning, particularly in patients with elevated CEA or predicted poor response. Interpatient variability highlights the need for biologically driven stratification, and future prospective studies integrating molecular profiling are warranted. CEA-based models may help identify candidates for organ-preserving strategies, but large multicenter trials are required before routine clinical implementation.

## Figures and Tables

**Table 1:** Quirke’s level of TME quality [12].

TME Quality Grade	
Complete	Intact mesorectum with a smooth surface; no defects deeper than 5 mm; no coning; and circumferential resection margin (CRM) appears intact
Near Complete	Moderate irregularity of the mesorectal surface; minor defects > 5 mm but not reaching the muscularis propria; and moderate coning may be present
Incomplete	Little bulk to the mesorectum; deep defects reaching the muscularis propria; marked coning; and irregular CRM

**Table 2:** Multivariate analysis of TRG regarding treatment.

	Reference	OR	95%CI		*p*-Value
Age (years)		0.97	0.92	1.02	0.302
Sex	Male	2.16	0.88	5.29	0.1
BMI		1.05	0.94	1.17	0.363
Tumor Location	Distal	0.86	0.35	2.1	0.741
Treatment	CRT	2.78	1.13	6.83	0.026

**Table 3:** CEA as a predictive marker of TRG.

		Response		OR (95% CI)	*p*-Value
		Good (0–1)	Bad (2–3)		
CEA levels	<5	34 (45.3%)	41 (54.7%)	4.15 (1.1–15.5)	0.032
	>5	3 (16.7%)	15 (86.3%)		

**Table 4:** Multivariate analysis of TRG (2–3) regarding CEA.

	Reference	OR	95% CI		*p*-Value
Age		1.03	0.97	1.09	0.297
Sex	female	2.71	1.04	7.07	0.042
BMI		0.93	0.83	1.04	0.209
Tumor Location	distal	1.50	0.58	3.90	0.406
Treatment	TNT	2.68	1.05	6.83	0.039
CEA	<5	6.33	1.38	29.02	0.018

## Data Availability

The data that support the findings of this study are available from the corresponding author upon reasonable request.

## Abbreviations

The following abbreviations are used in this manuscript:

TNT	Total Neoadjuvant Treatment
CRT	Chemoradiation
pCR	Pathological Complete Response
TRG	Tumor Regression Grade
PPV	Positive Prognostic Value
NPV	Negative Prognostic Value
CEA	Carcinoembryonic Antigen
TME	Total Mesorectal Excision
CRM	Circumference Resection Margin
